# Treatment with Distinct Antibiotic Classes Causes Different Pulmonary Outcomes on Allergic Airway Inflammation Associated with Modulation of Symbiotic Microbiota

**DOI:** 10.1155/2022/1466011

**Published:** 2022-06-22

**Authors:** Gregório Grama Cavalcante, Anna Gabriella Guimarães, Camila Pereira Queiroz-Glauss, Marcela Helena Gonçalves Pereira, Angélica Samer Lallo Dias, Laila Sampaio Horta, Jamil Silvano de Oliveira, Silvia Dantas Cangussú, Paula Prazeres Magalhães, Remo Castro Russo, H. C. Santiago

**Affiliations:** ^1^Department of Biochemistry and Immunology, Institute of Biological Science, Federal University of Minas Gerais (UFMG), Belo Horizonte, Brazil; ^2^Department of Microbiology, Institute of Biological Science, Federal University of Minas Gerais (UFMG), Belo Horizonte, Brazil; ^3^Laboratory of Experimental Pathophysiology, Department of Biological Sciences, Institute of Exact and Biological Sciences Center of Research in Biological Sciences, Federal University of Ouro Preto (UFOP), Ouro Preto, Brazil; ^4^Laboratory of Pulmonary Immunology and Mechanics, Department of Physiology and Biophysics, Institute of Biological Science, Federal University of Minas Gerais (UFMG), Belo Horizonte, Brazil

## Abstract

**Background:**

Asthma is a chronic pulmonary disease that affects about 300 million people worldwide. Previous studies have associated antimicrobial use with allergies, but the real impact of antibiotics on asthma is still elusive. We investigated the potential impact of amoxicillin (Amox), trimethoprim/sulfamethoxazole (TMP/SMX), and metronidazole (Metro) in a murine model of OVA-induced allergic airway inflammation.

**Methods:**

BALB/c mice received three cycles of 7 days of antibiotics in drinking water followed by 7 days washout and were sensitized i.p. with OVA/Alum at days 0 and 14. After the end of the last antibiotic washout, the mice were challenged with aerosolized OVA. Pulmonary parameters were evaluated, and serum, BAL, and feces were collected for analysis.

**Results:**

Amox- and TMP/SMX-treated animals displayed more severe allergic airway inflammation parameters with increased airway hyperresponsiveness, reduced lung alveolar volume, and increased levels in BAL of IL-4 and IL-6. In contrast, Metro-treated mice showed preserved FEV-50, decreased lung inflammation, and higher levels of butyrate and propionate in their feces. Metro treatment was associated with increased OVA-specific IgA in serum. BAL microbiota was abundant in allergic groups but not in nonallergic controls with the Amox-treated group displaying the increased frequency of *Proteobacteria*, while Metro and TMP/SMX showed increased levels of *Firmicutes*. In the gut, we observed the enrichment of *Akkermansia muciniphila* associated with reduced airway inflammation phenotype in the Metro group, even after the recovery period.

**Conclusion:**

Our data suggest that different antibiotic treatments may impact the course of experimental allergic airway inflammation in diverse ways by several mechanisms, including modulation of short-chain fat acids production by intestinal microbiota.

## 1. Introduction

Asthma is an allergic airway disease that affects more than 300 million people worldwide, being an important cause of life years with disability, especially in children (1). It consists of reversible bronchial obstruction triggered by airway hyperresponsiveness, causing shortness of breath, wheezing, and chest tightness (2). Allergic asthma is characterized by a Th2 type of immune response, marked by increased IL-4, IL-5, and IL-13 production, with systemic allergen-specific IgE and local eosinophil infiltration in the lungs (3), and also by a significant impact on the patient's microbiome (4).

Self-microbiota diversity can impact the individual's immunity, especially during the developmental period of life. For example, the “hygiene hypothesis” postulates that the loss of microbial taxa during the early infancy or the lack of exposure to infectious diseases is linked to impaired maturation of the immune system, making children more prone to allergies and other inflammatory diseases (5–7). In addition, the lifestyle exposed to the natural environment and a variety of microorganisms seems to protect against allergies and other inflammatory diseases when compared to the lifestyle of big cities with contact with limited environmental taxa (8, 9). Of note, children born by vaginal delivery, who are firstly colonized by microorganisms present in the vaginal canal, display a 20% reduction in asthma development when compared to children born by cesarean section, who are primarily colonized by the microbiota present in the skin from the healthcare providers (10).

Antibiotic use is among the most common mechanisms that disrupt the microbiome of an individual towards a less diverse microbial community, which may cause lifelong consequences (11–13), and early infancy seems a critical period of exposure (4, 14–18). It is known that the gut microbiome can produce several metabolites that are useful to the host, including vitamins and short-chain fat acids (SCFA) like acetate, propionate, and butyrate, with biological properties, including anti-inflammatory activity (11, 19). However, although many reports highlight the detrimental effects of early-life antibiotic use on child health (13, 16, 20, 21), there are doubts if the link between antibiotic use and allergies is clear. For example, conditions associated with allergy development and manifestation may render children more susceptible to infections early in life causing an increased use of antibiotics and bringing an important bias to the study design, a phenomenon known as reverse causation (22, 23). In addition, although dysbiosis caused by antibiotic use has been often associated with the development of allergies, other studies have found that neonatal colonization with specific bacteria as early as one month of age can predict future asthma development (24, 25), implying other elements besides antibiotic use in dysbiosis-related asthma. All these factors raise the complexity to reach a definitive conclusion on the antibiotic-allergy interface using human data.

In this study, we investigated the impact of antibiotic use during antigen sensitization in experimental allergic airway inflammation. We used the most prescribed antibiotics in pediatrics, i.e., amoxicillin (Amox), trimethoprim/sulfamethoxazole (TMP/SMX), or metronidazole (Metro) (26, 27), to induce dysbiosis during the sensitization with OVA interspersed with resting periods to allow microbiota recovery. Using a murine model improved the control of environmental, diet, and genetic cofounders allowing better observation of the antibiotic and its associated microbiota perturbation on the development of allergic airway inflammation. Our results indicate that some antibiotics, like Amox and TMP/SMX, have detrimental effects on allergic sensitization. In contrast, others, like Metro, can show beneficial effects on allergic sensitization.

## 2. Materials and Methods

### 2.1. Animals

Specific pathogen-free female BALB/cAnNCrl mice (21 days old) were obtained at Biotério Central of the Federal University of Minas Gerais and kept under temperature control, 12-hour light/dark cycle, and ad libitum supply of filtered water and autoclaved food. The local animal ethical committee approved all procedures (CEUA-UFMG, Brazil, protocol 25/2012).

### 2.2. Experimental Design

**Figure 1:**
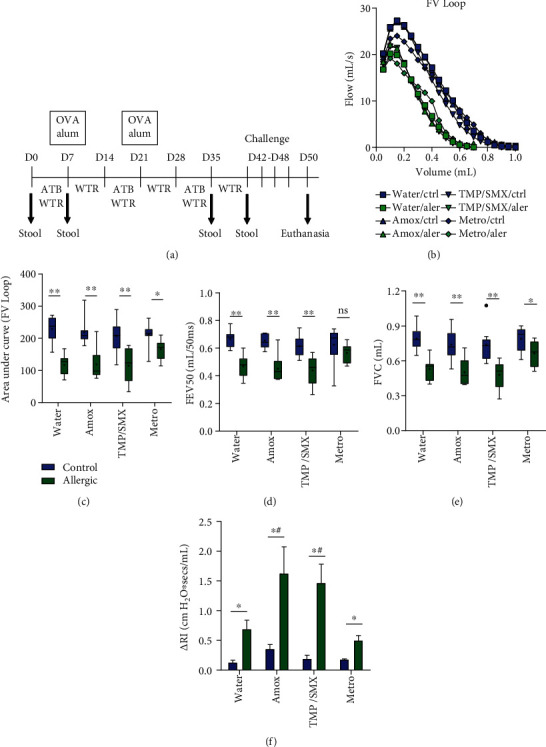
Different antibiotic treatments have diverse impact on asthma sensitization and development. (a) Experimental design. BALB/c animals were treated with antibiotics followed by a water resting period for 3 cycles. After the first two antibiotic treatments, animals were sensitized with OVA i.p. and, after the last resting period, challenged with autolyzed OVA to induce asthma. Animals were submitted to invasive plethysmography 48 h after the last challenge. (b) Flow-volume curve of allergic (green) and nonallergic controls (blue). (c) Area under the flow-volume curve. (d) Forced expiratory volume (FEV) in the first 50 ms and (e) forced vital capacity (FVC). Floating bars are Turkey representations with horizontal lines indicating means. (f) Delta airway resistance (RI) after i.v. injection of methacholine subtracted from parameters obtained before injection. Results are a pool of two independent experiments performed. *N* = 5 − 10 animals per group. Comparison between allergic and controls in the same treatment was performed by the Mann–Whitney test, and *P* < 0.05 is indicated by an asterisk (^∗^); differences between treatment groups and water-treated group were performed by the Kruskal-Wallis test, and *P* < 0.05 is indicated by a pound sign (#).

The mice were divided into four allergic and nonallergic group pairs receiving antibiotic treatment in the drinking water: (1) amoxicillin (Amox) at 50 mg/kg; (2) trimethoprim/sulfamethoxazole (TMP/SMX) at 24/120 mg/kg; (3) metronidazole (Metro) at 40 mg/kg; and (4) water-only control. The antibiotic doses were chosen based on literature (28–30). Treatment consisted of 7 days with antibiotics in the drinking water followed by 7 days of washout (resting), repeated for 3 cycles (Figure 1(a)). Washout periods of 7 days were chosen to allow at least 5 half-lives of the drug with the higher elimination period (metronidazole, 4 hours) and to allow reconstitution of the microbiota. Ovalbumin (OVA) allergic airway inflammation model was performed as described (31), with minor modifications. Allergic groups received intraperitoneal injections with 10 *μ*g of grade II ovalbumin (Sigma-Aldrich, Missouri, USA) with Alhydrogel® (Brenntag, Denmark) as adjuvant after the first cycle of antibiotics and 14 days later (Figure 1(a)). Nonallergic groups received injections with Alhydrogel® only. At the end of the third recovery period, the animals were challenged in a nebulization chamber with aerosolized 1% ovalbumin suspension for 4 times on alternate days for 20 minutes each session.

### 2.3. Spirometry Tests

Forty-eight hours after the last challenge, animals were anesthetized with an association of xylazine (12.5 mg/kg) and ketamine (100 mg/kg) subcutaneously and subjected to invasive spirometry (Buxco Research Systems©, USA). We determined FEV50, FVC, resistance, and FV loop, as previously described (32), and after the first cycle of pulmonary tests, the mice received 1 mg/kg of methacholine intravenously to determine airway hyperresponsiveness in 20 seconds.

### 2.4. ELISA for IgG1, IgA, and IgE in Serum and BAL

Polystyrene plates were coated with ovalbumin solution at 5 *μ*g/mL in carbonated coating buffer at 4°C overnight. On the next day, the plates were washed (PBS 1X+0.05% Tween®20) and blocked (PBS 1X+0.25% casein) at room temperature for 2 hours. For IgG detection, the serum samples were diluted at 1 : 1000 and BAL at 1 : 20. For IgA, the serum samples were diluted at 1 : 10 and BAL at 1 : 20. For IgE, both serum and BAL samples were diluted at 1 : 20. Samples were incubated at 4°C overnight. Plates were then washed and incubated with biotinylated anti-mouse IgG1 (Thermo-Scientific, USA), IgA (eBioscience, USA), or goat anti-mouse IgE (Abcam, USA) antibodies for 1 hour at room temperature. Plates were washed and incubated with streptavidin-conjugated to peroxidase (IgG and IgA) (Sigma-Aldrich), for 1 hour at room temperature. The reaction used OPD and was stopped with sulfuric acid. Colorimetric absorbances were measured at 492 nm.

### 2.5. Cytokine Bead Array Th1/Th2/Th17 of the BAL Supernatant

The assay for the detection of the cytokines IL-2, IL-4, IL-6, IFN-*γ*, TNF, IL-17A, and IL-10 through flow cytometry was performed on BAL supernatants. The test was performed according to the manufacturer's instructions (BD Biosciences, San Jose, USA).

### 2.6. DNA Extraction and Sequencing Analysis

Mice's feces, pooled from animals in each cage, were collected before the antibiotic treatment (day 0), after the end of the first cycle (day7), after the end of the third cycle (day 35), and after 7 days of the end of the third cycle (day 42), before challenge (Figure 1(a)). The feces were immediately frozen in a -80°C freezer for subsequent extraction of bacterial DNA. The DNA of the samples was extracted using the QIAamp DNA stool mini kit (Qiagen, Germany), following the manufacturer's instructions. DNA extraction from the BAL was performed on alcohol-acid affinity and resuspended with 30 *μ*L of DNase/RNase-free water.

### 2.7. 16S rDNA Gene Sequencing and Data Analysis

16S rDNA sequencing was carried out by Neoprospecta (Florianópolis, Santa Catarina, Brazil) according to the company's protocols using primers 314F-806R, which target the hypervariable regions V3-V4, allowing the detection of microorganisms from the Bacteria and Archaea domains (33) on the MiSeq Illumina platform. The DNA sequences were individually filtered for quality allowing a maximum of 1% of accumulated error. The sequences approved by the screening process were grouped into clusters. They were used for taxonomic identification by comparison with a database (NeoRef, Neoprospecta) using the BLAST software (NIH). Analyses of relative abundance and construction of heatmaps (normalized log10 abundance of each OTU in each sample) were performed using the R program (https://www.r-project.org-R, Core Team 2014), employing ggplot2 and heatmap packages, respectively.

### 2.8. Histopathological Analysis of the Lungs

Paraffin-embedded lungs were stained with hematoxylin-eosin (HE) and periodic acid Schiff (PAS). Slides were photographed in a 20X and 40X objective using the ABX 35 OLIMPUS® microscope and Image-Pro Express version 4.0 program to Windows (Media Cybernetics, Bethesda, MD, USA). To estimate the area of inflammation, eighteen fields from each HE slide were randomly chosen on the periphery of intrapulmonary bronchi and blood vessels, photographed at a 20X objective, reaching a total area of 75910.81 *μ*m^2^. To estimate mucus production, five to eighteen fields of the conducting intrapulmonary airways that presented mucociliary epithelia with similar diameters were selected in each PAS slide and photographed with a 40X objective. Morphometric analyses were performed by using ImageJ 2 (http://rsb.info.nih.gov/ij/) (34). The analysis of the volume density of alveolar spaces (Vv [a]) and alveolar graves was performed through stereology as previously described (35).

### 2.9. Dosage of SCFA

The dosage of organic acids was performed as previously reported (36), with adaptations. After the third washout, 200 mg of feces from each animal was collected in microtubes to which 1.2 mL of ultrapurified water at pH 2.5 was added. The feces were then macerated for 30 seconds in an electric tissue homogenizer. After 10 minutes at room temperature, the tubes were centrifuged at 13200 RPM for 30 minutes. Supernatants were collected and frozen at -80°C for further analysis. Samples were loaded onto a Nexera UHPLC (Shimadzu) with a SUPELCOGEL C6-610H column (Sigma-Aldrich) in a volume of 40 *μ*L per chromatographic run. Regarding the characteristics of the run, the mobile phase is composed of sulfuric acid 0.01 N, a temperature of 40°C, and a flow of 0.6 mL/min. The identification of the compounds was made by comparing the retention time in relation to acetate, propionate, and butyrate standards.

### 2.10. Data Analysis

The data obtained with the experiments were analyzed using the GraphPad Prism 6 program (La Jolla, USA). The Mann–Whitney test was performed to compare the pair control-allergic groups within the same treatment, and statistically significant differences (*P* < 0.05) were indicated by an asterisk (^∗^). For comparison between different antibiotic treatment groups, Kruskal-Wallis was used, and statistically significant differences (*P* < 0.05) were indicated by the pound sign (^#^). Except when specified, the number of mice used in the experiments was between 5 and 15.

## 3. Results

### 3.1. Different Antibiotic Classes/Spectrums Have a Distinct Impact on Pulmonary Dysfunction during Allergic Airway Inflammation in Mice

We evaluated the impact of antibiotic treatment during sensitization in experimental ovalbumin- (OVA-) induced allergic airway inflammation. BALB/c mice were treated with water only or water containing amoxicillin (Amox), trimethoprim/sulfamethoxazole (TMP/SMX), or metronidazole (Metro) during OVA sensitization. After antibiotic treatment, allergic airway inflammation was induced by OVA nebulization (Figure 1(a)). Allergic mice showed increased pulmonary dysfunction, as observed by reduced airway flow (Figures 1(b) and 1(c)), except for the Metro group, which showed normal FEV50, but reduced FVC (Figure 1(d)). Despite improving some respiratory parameters, the allergic Metro group displayed airway hyperreactivity triggered by methacholine comparable to the water control group (Figure 1(f)). In contrast, Amox and TMP/SMX groups presented higher methacholine-induced airway resistance (Figure 1(f)), while presenting typical allergic flow-volume curves, FVC, and FEV50 comparable to water control allergies (Figures 1(b)–1(e)).

### 3.2. Different Antibiotics Have Distinct Effects on Allergic Airway Inflammation Immunopathology in Mice

**Figure 2:**
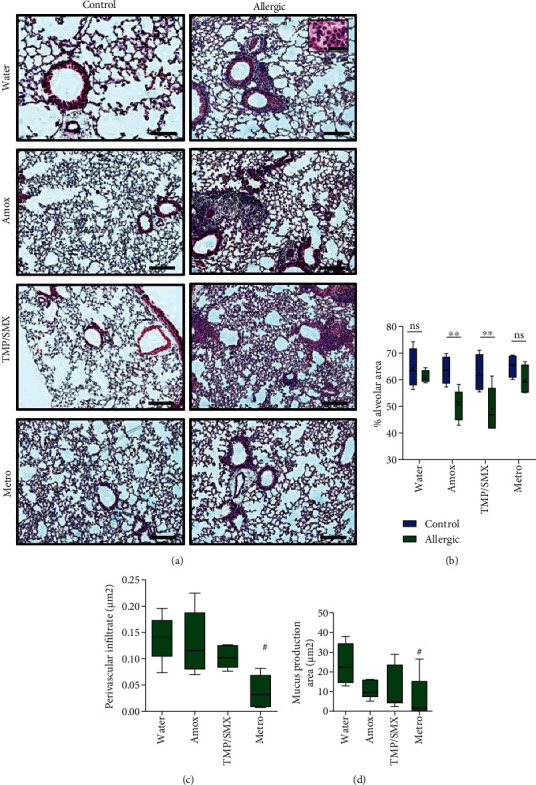
Amox and TMP/SMX promote loss of alveolar area, while Metro decreases inflammatory infiltrate and mucus production in allergic animals. BALB/c mice treated with different antibiotics during OVA sensitization were challenged on 4 alternate days with aerosolized OVA. Histopathology was evaluated 48 hours after the last challenge. H&E (a) and PAS staining (not shown) were performed. (b) The volume of alveolar area calculated by stereology, (c) area of perivascular infiltrate, and (d) area of mucus production were all performed according to the description in methods. *N* = 5 − 6 animals per group. Experiments are representative of one from two performed. Floating bars are Turkey representations with horizontal lines indicating means. (b) Differences between controls and allergic animals were evaluated by the Mann–Whitney test, and *P* < 0.05 was indicated by asterisks (^∗^). Differences between treatments were evaluated by the Kruskal-Wallis test, and *P* < 0.05 was indicated by the pound sign (^#^).

**Figure 3:**
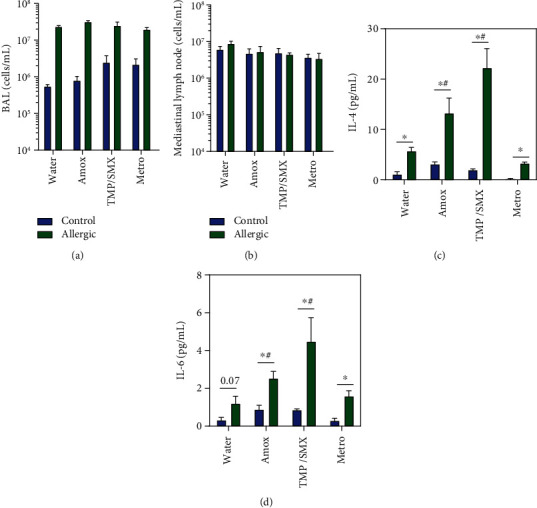
Amox and TMP/SMX induced higher levels of IL-4 and IL-6 in BAL of allergic animals. BALB/c mice treated with different antibiotics during OVA sensitization were challenged on 4 alternate days with aerosolized OVA. Cell count in (a) BAL and (b) draining mediastinal lymph nodes were evaluated 48 hours after the last challenge. (c) IL-4 and (d) IL-6 were evaluated in BAL by CBA. Bars represent mean ± SE. Experiment representative of two was performed with similar results. *N* = 5 per group. Differences between controls and allergic animals were evaluated by the Mann–Whitney test, and *P* < 0.05 was indicated by asterisks (^∗^). Differences between treatments were evaluated by the Kruskal-Wallis test, and *P* < 0.05 was indicated by the pound sign (^#^).

Allergic animals displayed a typical peribronchial mixed infiltrate rich in lymphocytes and eosinophils (Figure 2(a)). Interestingly, while Amox and TMP/SMX groups showed more intense lung cellular infiltrates, the Metro group showed decreased levels of inflammation when compared to the water group (See Supplemental Figure 1 for higher magnifications). These findings were reflected in the lung alveolar area and quantification of peribronchial inflammation. For example, the use of Amox and TMP/SMX caused an important reduction in lung alveolar area in allergic mice, suggesting a detrimental effect of these drugs on parenchyma infiltration associated with allergic airway inflammation (Figure 2(b)). In contrast, the Metro-treated allergic group presented decreased peribronchial cellular infiltrate (Figure 2(c)) and lower levels of mucus deposition upon OVA challenge (Figure 2(d)) when compared with the allergic water-treated group. Interestingly, antibiotic treatment did not change the cellular recovery in BAL and mediastinal lymph nodes of the allergic animals (Figures 3(a) and 3(b)). Although, the profile of the cellular infiltrate was not accessed, we could notice that the cytokine balance has deviated. Treatment with Amox and TMP/SMX was associated with higher levels of IL-4 and IL-6 on BAL of allergic mice (Figures 3(c) and 3(d)), but not IL-2, IFN-*γ*, TNF, IL-17A, and IL-10 (data not shown).

### 3.3. Metro Treatment Raises Anti-OVA IgA in the Serum from Allergic Mice

**Figure 4:**
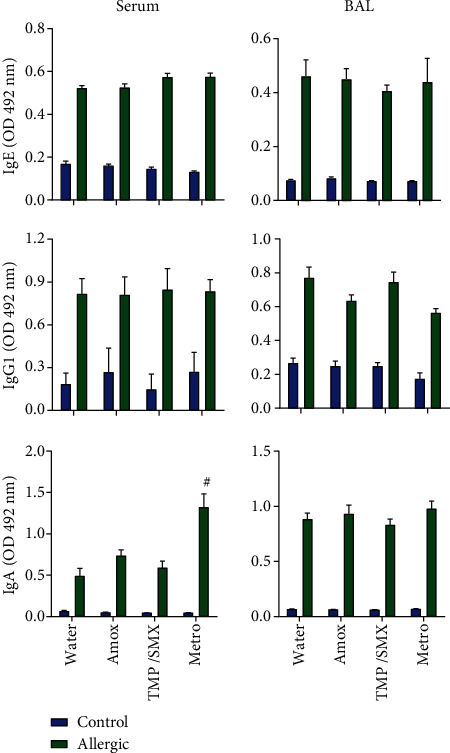
Metronidazole increases OVA-specific IgA in BAL of allergic mice. BALB/c mice treated with different antibiotics during OVA sensitization were challenged on 4 alternate days with aerosolized OVA. 48 hours after the last challenge, animals were euthanized, and BAL and serum were collected for measurement of OVA-specific IgE, IgG1, and IgA by ELISA. Bars represent mean ± SE. Experiment representative of two performed with similar results. *N* = 4 − 5 per group. Differences between water-treated allergic animals and antibiotic-treated allergic groups were evaluated by the Kruskal-Wallis test, and *P* < 0.05 was indicated by the pound sign (^#^).

Allergic mice displayed OVA-specific IgE, IgG1, and IgA that could be detected in both serum and BAL. Antibiotic treatment did not modulate IgE or IgG1 anti-OVA. On the other hand, treatment with Metro induced higher levels of IgA in serum but not in BAL (Figure 4).

### 3.4. Antibiotic-Treated Mice Have a Different Microbiota Profile

**Figure 5:**
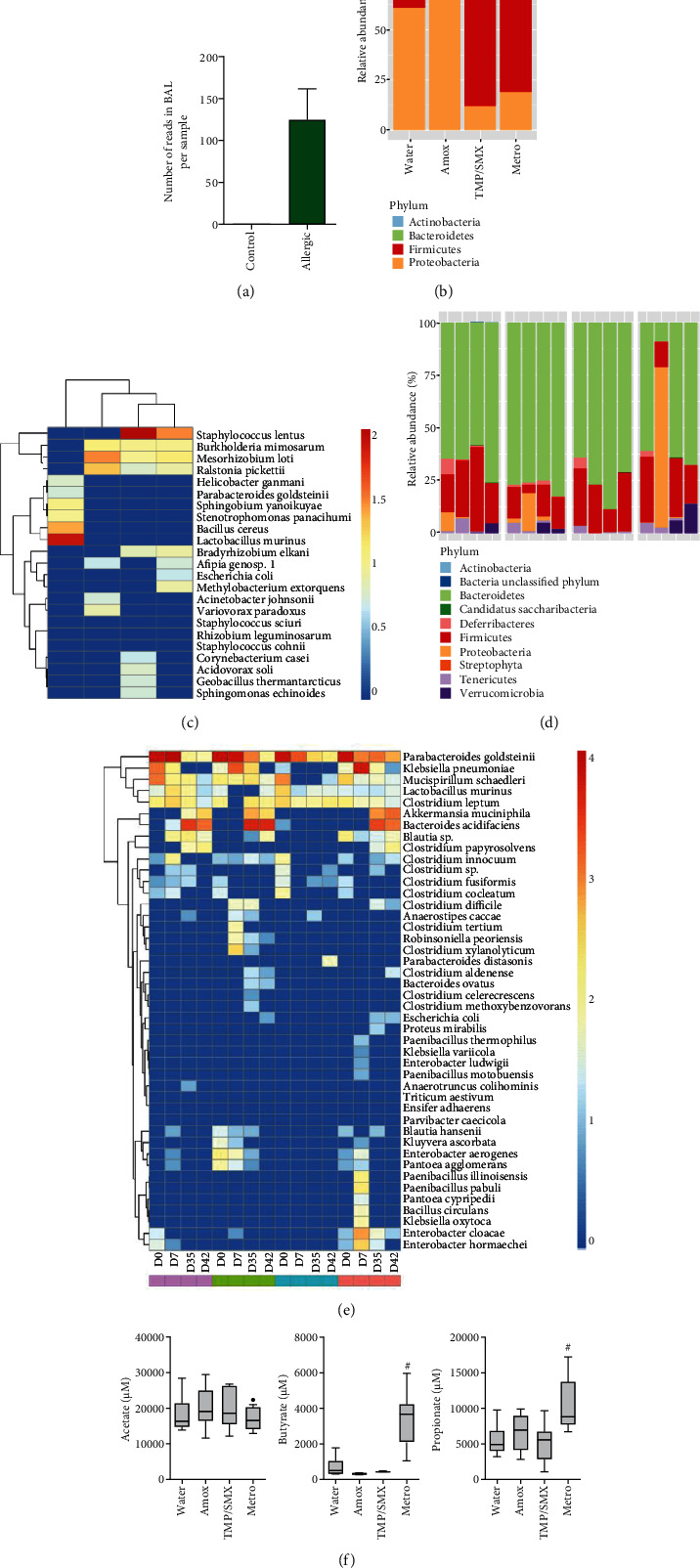
Antibiotic treatments altered gut and BAL microbiota and SCFA in feces. BALB/c mice were treated with different antibiotics during OVA sensitization. At days 0 (before treatment), 7 (after the first antibiotic cycle), 35 (after the third antibiotic cycle), and 42 (after the last resting period and before challenge), animals had feces collected for DNA extraction and 16S sequencing. BAL was collected on day 50 (48 hours after the OVA challenge) and DNA extracted for 16S sequencing as well. On day 42 (after the last resting period and before the challenge), animals had feces collected for measurement of SCFAs. (a) Number of OTU reads in BAL comparing allergic and nonallergic animals. Bars represent means ± SE of 12 allergic animals and 3 nonallergic controls pooled from 2 independent experiments. (b) Phyla and (c) OTUs identified in BAL of allergic animals. (d) Phyla and (e) OTUs identified in feces. (f) Concentration of SCFA in mice stool. Floating bars are Turkey representations with horizontal lines indicating means. Results are a pool of two independent experiments performed. *N* = 9 − 10 animals per group. Differences between allergic antibiotic-treated animals and allergic water-treated animals were evaluated by the Kruskal-Wallis test, and *P* < 0.05 was indicated by the pound sign (^#^).

Allergic airway inflammation induced higher numbers of microbial DNA reads in the BAL when compared to nonallergic controls (Figure 5(a)). However, no significant differences were found between the antibiotic treatments. We could observe that the water allergic group presented balanced levels between *Proteobacteria* and *Firmicutes* in BAL (Figure 5(b)). On the other hand, animals treated with Amox presented higher levels of *Bacteroidetes* when compared to the water group, with phylum *Proteobacteria* dominance. In contrast, allergic mice treated with Metro, and surprisingly also with TMP/SMX, presented higher *Firmicutes* and lower *Proteobacteria* levels (Figure 5(b)). Of note, antibiotic treatment changed the balance of OTUs recovered from the BAL. For example, *Helicobacter ganmani*, *Parabacteroides goldsteinii*, *Sphingobium yanoikuyae*, *Stenotrophomonas panacihumi*, *Bacillus cereus*, and especially *Lactobacillus murinus* were enriched in the TMP/SMX-treated animals (Figure 5(c)). In contrast, Metro treatment caused the enrichment of *Corynebacterium casei*, *Acidovorax soli*, *Geobacillus thermantarcticus*, and *Sphingomonas echinoides* (Figure 5(c)). Few OTUs were reduced when compared to the water-treated group. For example, most antibiotic treatments reduced the presence of *Bradyrhizobium elkanii* (except Metro), *Afipia* sp. (except Amox), *Escherichia coli*, and *Methylobacterium extorquens*. Importantly, *Staphylococcus lentus* seemed to be particularly associated with the allergic airway inflammatory phenotype induced by the different antibiotics. For instance, while Metro-treated mice displayed increased OTU frequencies of *S. lentus* OTU, reduction was observed with Amox and TMP/SMX treatment in the BAL of allergic mice compared to the water-treated group (Figure 5(c)).

In the gut microbiota, we observed dominance of *Bacteroidetes* and *Firmicutes* at D0 (before antibiotic treatment) in all groups, which also showed the variable presence of *Proteobacteria*, Tenericutes, and Deferribacteres (Figure 5(d)). On days 7 and 35 (after the first and third antibiotic treatment cycles, respectively), we could observe substantial microbiota perturbation with decreased abundance of *Firmicutes* OTUs with Amox, TMP/SMX, and Metro treatments. An important expansion of *Proteobacteria* (day 7) and *Verrucomicrobia* (day 42) in animals treated with Amox and Metro, respectively, was also observed. No alteration in gut phyla seemed particularly associated with the allergy sensitization or allergy induction.

Although we could observe strong microbiota resilience in the gut following antibiotic treatment-induced dysbiosis, some microbiota scars could be observed. For example, when compared to the water-treated group, *Blautia* sp. was found enriched or at least maintained in the gut of Metro-treated groups and strongly reduced in the gut of mice that received Amox (except for day 42 when it showed recovery) and TMP/SMX (all time points) when compared to water-treated mice (Figure 5(e)). On the other hand, mice treated with Metro, which showed enrichment of *Verrucomicrobia* (Figure 5(d)), showed a sustained enrichment of *A. muciniphila* at day 42. Mice treated with TMP/SMX kept high levels of *P. goldsteinii* in all points analyzed, a species common in all other groups postweaning but waned as mice entered adulthood (Figure 5(e)).

### 3.5. Metronidazole-Treated Mice Have Elevated SCFA in Stools

Short-chain fat acids (SCFA) are an important metabolite of the gut microbiota for the host, which are associated with modulation of the host's metabolism and immune response (19), including modulation of asthma (37, 38). We also sought to evaluate if antibiotic-induced differences in microbiota and allergy phenotype were associated with gut modulation of SFCA in feces. We observed that animals treated with the Metro group presented higher levels of butyrate and propionate, but not acetate, in the feces, when compared to the control group (Figure 5(f)).

## 4. Discussion

There is an important debate about the impact of antibiotic use on asthma and other allergic diseases. For example, while antibiotic use is more common among asthmatic patients, they are also more prone to respiratory infections, which is a critical bias (39). Protopathic bias, also called reverse causation, can hinder this association since physicians can prescribe those drugs for the first asthmatic or wheeze symptoms that can be mistaken for respiratory infections (40). Since asthma is a multifactorial disease, which makes the control of variables in human studies very difficult, the use of an experimental model offers better monitoring of all variables and can provide definite answers.

Using a murine model airway allergy, we could determine that antibiotics alter the course of airway inflammation. However, the impact on airway allergy seems to be dependent on the antibiotic class/spectrum. For example, Amox and TMP/SMX treatments showed a negative impact on experimental allergic airway inflammation, increasing airway hyperresponsiveness, and the presence of proinflammatory cytokines in the BAL. On the other hand, the use of Metro was associated with attenuated effects of airway inflammation and mechanical dysfunction, suggesting preservation of lung capacity.

The mechanisms seemed to vary for the antibiotic classes. For example, Metro-treated allergic mice also displayed increased OVA-specific IgA in the blood, but surprisingly, not in BAL. IgA deficiency (IgAD), total or partial, has been associated with increased asthma prevalence and other respiratory allergic diseases (41–43). Low serum levels of IgA are associated with severe asthma, worse pulmonary function (44), and increased risk of respiratory infection (43), especially in the asthmatic population (45). Interestingly, serum IgA may impact allergy by supposedly neutralizing antigens at the site of allergy, e.g., directly in the lungs, or by modulating intestinal microbiota. For example, IgA bound to intestinal bacteria is different between healthy and asthmatic children. Allergic children show decreased IgA binding to *Bacteroides* genus but increased binding to *Firmicutes*, even presenting similar IgA serum levels to healthy controls (46). In this study, we evaluated only OVA-specific IgA. However, the exact mechanism induced by Metro that caused increased levels of OVA-specific IgA is still elusive.

A healthy gut microbiota nurtures a suitable environment for host homeostasis (19). Microbiota disorders are related to chronic diseases (5, 6, 47, 48). The microbiota is essential for immune system activity and is deeply linked to changes in the allergic phenotype. For example, germ-free animals display higher susceptibility to experimental airway hypersensitivity, which is attenuated by microbiota colonization (49, 50). Likewise, microbiota disturbance changes the phenotype of asthma. Animals treated with vancomycin during the neonatal period develop more severe asthma-like symptoms than nontreated mice; in contrast, streptomycin shows little impact (51). Our findings corroborate these data, in which antibiotic-specific effects on microbiota may imply different outcomes of airway allergy. We found that the antibiotics temporarily altered the gut microbiota, which could recover spontaneously after a resting period of only 7 days. However, since we sensitized mice during antibiotic-induced dysbiosis, we observe the impact on antigen sensitization and its implications on allergic airway inflammation even after the microbiota recovery period.

We also found that allergic airway inflammation induced by OVA can profoundly impact the microbial colonization of the lungs. While nonallergic animals showed undetectable levels of 16S DNA in BAL, we found elevated 16S DNA in BAL of allergic mice. Recent metagenomics studies have demonstrated the presence of bacterial DNA in sites usually thought to be microbe-free, such as the amniotic fluid, blood, and the lower respiratory tract (52). However, we found that nonallergic mice were free of 16S DNA in the BAL, but allergic mice showed increased bacteria DNA levels. Accordingly, increased bacterial DNA of organisms such as *Firmicutes*, *Bacteroidetes*, and especially *Proteobacteria* was found in the airways of asthmatic patients compared to healthy individuals (53). We found that amoxicillin increased the abundance of *Proteobacteria* in the BAL compared to the allergic control group. *Bacteroidetes* are also detected as minor components of BAL microbiota in Amox- and TMP/SMX-treated groups. *Bacteroidetes* are a phylum of anaerobic Gram-negative bacteria present in the gut of mice and humans and considered a typical component of the lung microbiome (54). Notably, the treatment with Metro, which improved airway allergy parameters, also showed a high relative abundance of *Staphylococcus lentus* in the lungs, which may have probiotic benefits in murine models of OVA-induced allergy when administered intranasally (55).

Aside from the temporary dysbiosis caused by antibiotic treatment, we observed that some antibiotics may have left a microbiome scar in the stool. Metro-treated animals displayed enrichment, while TMP/SMX showed inhibition of *A. muciniphila*, which were kept even after treatment interruption. This bacterium has been shown to play protective roles in inflammatory diseases such as asthma (56). The levels of *Akkermansia* have also been reported to increase in an experimental model of colitis with *Clostridium difficile* (57) and anxiety (58) after treatment with metronidazole, which is also associated with improved disease condition. To our knowledge, this is the first report showing a decrease of *A. muciniphila* in the gut microbiome caused by TMP/SMX or Amox. Their reduced relative abundance is associated with metabolic syndrome, obesity, and diabetes (59). Likewise, *Blautia* sp., which in our data can refer to *Blautia* sp., *Roseburia* sp., or *Coprococcus* sp., belongs to the *Clostridium* XIVa group, an important producer of SCFA (60), was enriched in Metro-treated animals. Interestingly, most antibiotic treatment reduces SCFA production, such as acetate, butyrate, and propionate (61–65). However, metronidazole has been described to reduce (64) or not interfere (63) with SCFA levels. Some have reported that metronidazole may even increase lactate fecal levels (66). We found that treatment with metronidazole was associated with increased levels of *Blautia* sp., a bacteria involved in SCFA production, and also increased levels of propionate and butyrate, important modulatory SCFA. Indeed, SCFA are known for their systemic immunomodulatory role in reducing inflammation (67). SCFA act on various cell receptors like GPR41, GPR43, and aryl hydrocarbon receptor precursor (AhR), among others, modulating immunity (67). For example, SCFA can impact antibody production (68, 69), modulate Treg activation (70–72), and decrease inflammatory cell infiltrates, including allergic inflammation. For instance, propionate supplementation alleviates the allergic airway inflammation in mice (73). In addition, decreased fecal butyrate levels are associated with worse asthma-like and asthma manifestation in mice (74) and humans (63). Of note, *A. muciniphila*, another bacteria enriched by metronidazole in our treatment, cannot produce butyrate directly, but it can degrade mucin and generate metabolites useful as a source of these fatty acids by other bacteria. On the other hand, Amox-treated mice displayed higher levels of *C. difficile* OTU during sensitization, a bacterium associated with an increased risk of allergies development in childhood (75). Some antibiotics can also impact other microorganisms' phyla. For example, metronidazole can disturb symbiotic protozoa (76); however, this possible action was not evaluated in this manuscript despite being an additional mechanism that may bring light to some of the data that still require further investigation. Nevertheless, our data still indicate that treatment with antibiotics may have adverse effects on the natural microbiota with an impact on inflammatory diseases.

In conclusion, we demonstrated that antibiotics modulate the response to allergen sensitization and allergic airway inflammation development in mice. Our data support that antibiotics cause long-lasting alterations in the microbiota balance, which can interfere with the regulatory and proinflammatory axis of the immune system. Interestingly, while some antibiotics may have detrimental effects on allergic airway inflammation, others may offer positive consequences. In this sense, the irrational use of antibiotics is quite common worldwide and should be dealt with caution as they can reverberate in patients' health, possibly for many years through dysbiosis “scars.” Finally, this study opens perspectives on the selection of antibiotics that will be less detrimental to the patient's immunity, especially in those showing allergy-prone phenotype, and perhaps for the use of antibiotics to shift the microbiota balance towards a less inflammatory phenotype.

## Data Availability

The 16S rDNA gene sequencing data used to support the findings of this study have been deposited in the NCBI repository under the GenBank numbers MW234071-MW234136. The spirometry, pathology, and immunology data used to support the findings of this study are included within the article or supplementary data. Data not shown, including raw data and pathology slides photos, used to support the findings of this study are also available from the corresponding author upon request.

## Additional Points

*Key Messages*. (i) The use of antibiotics impacts the sensitization and development of allergic airway inflammation in a drug-dependent manner in controlled experimental settings. (ii) Amoxicillin and sulfamethoxazole/trimethoprim displayed a negative impact, while metronidazole improved allergic airway inflammation parameters. (iii) Immunological and microbiological mechanisms associated with allergic airway inflammation were modulated by antibiotic use. (iv) Antibiotics may be selected to modulate the immune system to decrease or increase the development of allergic diseases.

## Supplementary Materials

Supplementary Materials Different antibiotics produce divergent impacts on asthma development, while amoxicillin and trimethoprim/sulfamethoxazole worsen the pathology and respiratory parameters by selecting microbiota associated with inflammatory status; metronidazole selects microbiota associated with short-chain fatty acids that promote the regulation of experimental allergy.
