# Supplementary figures and images for: Treatment with Distinct Antibiotic Classes Causes Different Pulmonary Outcomes on Allergic Airway Inflammation Associated with Modulation of Symbiotic Microbiota
Source: J Immunol Res. 2022 Jun 22;2022:1466011. doi: 10.1155/2022/1466011 (PMC9242750; doi:10.1155/2022/1466011)

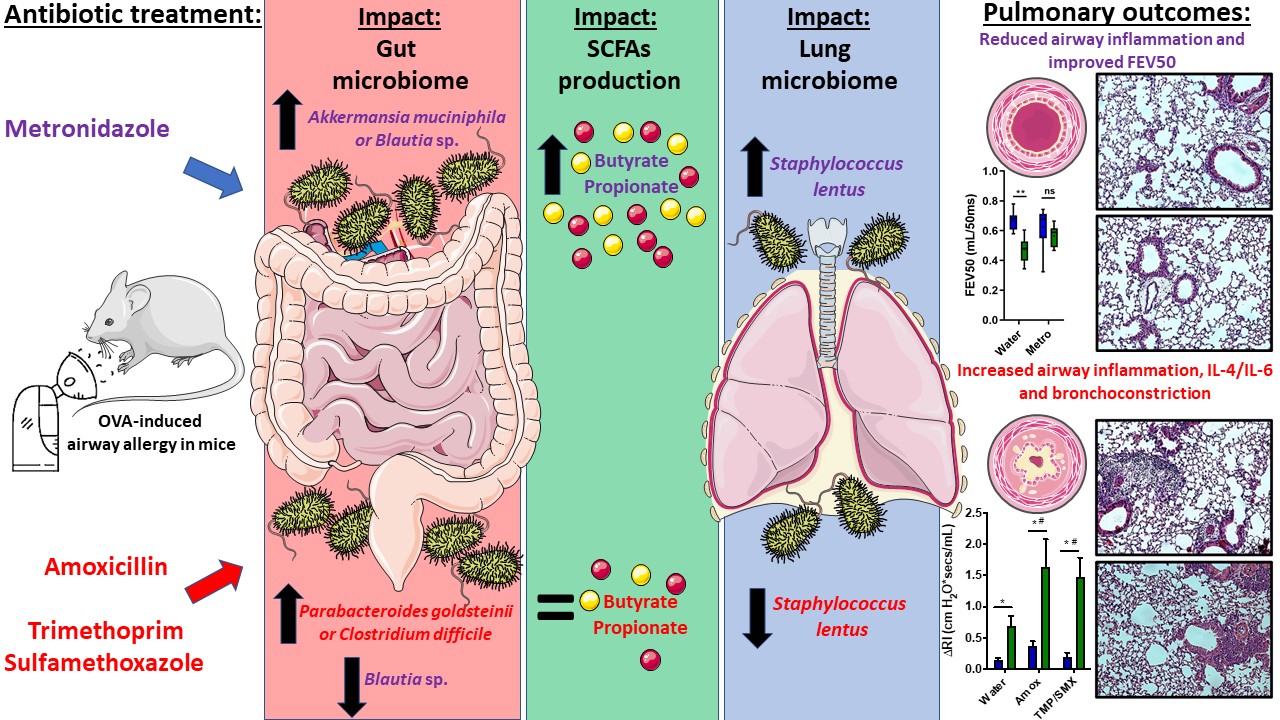

Supplement: Supplementary Materials — Different antibiotics produce divergent impacts on asthma development, while amoxicillin and trimethoprim/sulfamethoxazole worsen the pathology and respiratory parameters by selecting microbiota associated with inflammatory status; metronidazole selects microbiota associated with short-chain fatty acids that promote the regulation of experimental allergy. [file 1466011.f1.zip › Graphical Abstract.jpg]

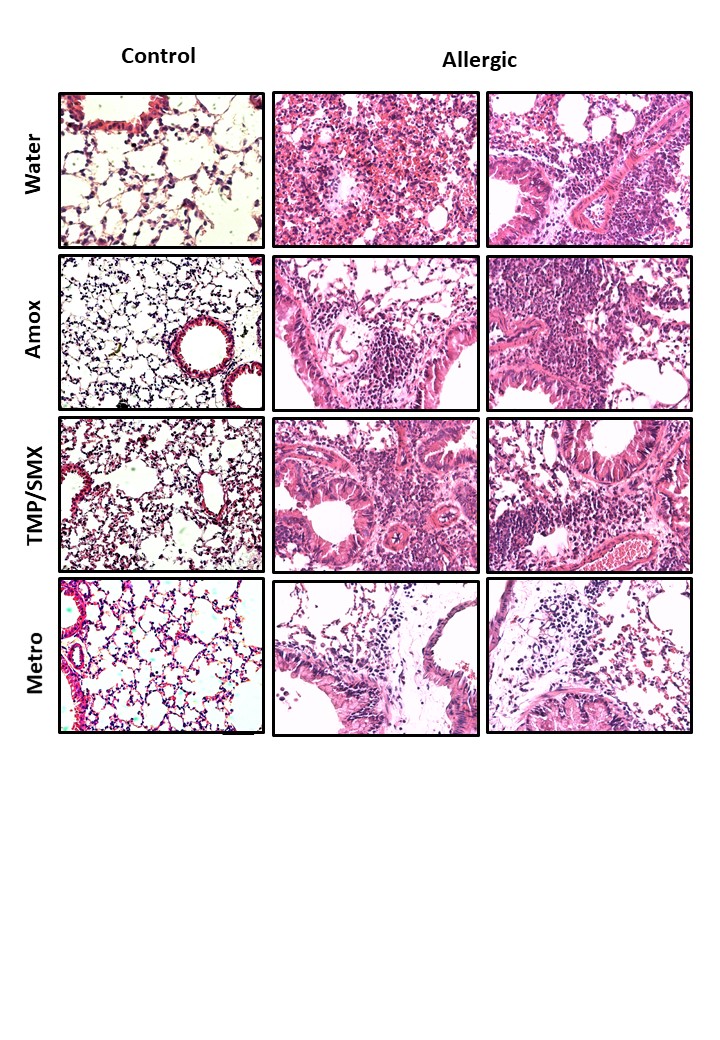

Supplement: Supplementary Materials — Different antibiotics produce divergent impacts on asthma development, while amoxicillin and trimethoprim/sulfamethoxazole worsen the pathology and respiratory parameters by selecting microbiota associated with inflammatory status; metronidazole selects microbiota associated with short-chain fatty acids that promote the regulation of experimental allergy. [file 1466011.f1.zip › Supplemental Figure 1.jpg]
